# Effects of multimerization on the temporal variability of protein complex abundance

**DOI:** 10.1186/1752-0509-7-S1-S3

**Published:** 2013-08-12

**Authors:** Antti Häkkinen, Huy Tran, Olli Yli-Harja, Brian Ingalls, Andre S Ribeiro

**Affiliations:** 1 Department of Signal Processing, Tampere University of Technology, P.O. box 553, 33101 Tampere, Finland; 2 Institute for Systems Biology, 1441 North 34th Street, Seattle, Washington 98103-8904, USA; 3 Department of Applied Mathematics, University of Waterloo, 200 University Avenue West, Waterloo, Ontario, Canada

## Abstract

We explore whether the process of multimerization can be used as a means to regulate noise in the abundance of functional protein complexes. Additionally, we analyze how this process affects the mean level of these functional units, response time of a gene, and temporal correlation between the numbers of expressed proteins and of the functional multimers. We show that, although multimerization increases noise by reducing the mean number of functional complexes it can reduce noise in comparison with a monomer, when abundance of the functional proteins are comparable. Alternatively, reduction in noise occurs if both monomeric and multimeric forms of the protein are functional. Moreover, we find that multimerization either increases the response time to external signals or decreases the correlation between number of functional complexes and protein production kinetics. Finally, we show that the results are in agreement with recent genome-wide assessments of cell-to-cell variability in protein numbers and of multimerization in essential and non-essential genes in *Escherichia coli*, and that the effects of multimerization are tangible at the level of genetic circuits.

## Introduction

Proteins regulate various cellular processes. There are several mechanisms responsible for regulating their numbers in cells, which act at various stages of protein production [1-4], activation [5], and degradation. A recent study has provided genome-wide information on protein numbers in *Escherichia coli *along with their cell-to-cell variability [6]. In total, 121 were classified as essential, while 894 were classified as non-essential. Addressing multimerization, it was found that 719 proteins are functional in a monomeric form, while 198 function in a dimeric form, 16 in trimeric, 47 in tetrameric, and the remaining in higher-order forms. Multimerization is likely to arise from the need for functionality, and such a need varies significantly between proteins. Some proteins are functional both in monomeric as well as in various multimer forms [7,8], while others are only functional in a specific form [9].

The process of multimerization, aside from being related to the functionality of the proteins, may also affect the dynamics of the processes that the proteins regulate. This is expected given that multimerization necessarily affects the mean numbers of functional proteins, the response times of the cell (e.g. to external signals), and the degree of correlation between RNA numbers and the corresponding functional protein complex numbers, i.e. the degree of control that transcription factors have on the protein complex numbers over time. These effects can be expected to propagate to the network level. For example, in genetic switches, where stochastic fluctuations in protein numbers determine, among other factors, the switching frequency [10,11], cooperative binding of the proteins enhances the range of conditions for which bistability is observed [12].

The dynamics of protein abundance depend on the transcriptional and translational dynamics as well as on the kinetics of degradation of RNA and proteins. Therefore, to assess the effects of multimerization on the dynamics of gene expression and of genetic circuits one needs to model the kinetics of these processes in detail. The RNA production rate of a gene is mainly controlled during the process of transcription initiation, at the promoter region (see [1] for a review). Recent in vivo measurements of the intervals between the production of individual transcripts [13,14] suggest that, under normal growth conditions, there are two to three significant rate-limiting steps at the initiation stage that, aside from determining the mean rate of production, also determine the degree of noise in the process of RNA production. In prokaryotes, these observations relate directly to protein copy numbers, which tend to follow closely those of RNA [15]. To account for the stochasticity and the rate limiting steps of the underlying steps in the process of gene expression, we use the delayed stochastic modeling strategy [16] to drive the dynamics of the models, as it allows the use of non-Markovian dynamics to model the non-instantaneous processes underlying transcription and translation [17]. The parameters used in the models are extracted from live, single-cell, single-molecule measurements [6,13,14].

Using the modeling and simulation techniques mentioned above, along with realistic parameter values, we investigate the consequences of multimerization on mean numbers and fluctuations and on the response time of functional protein complex numbers to external signals. Further, we investigate whether these effects have tangible consequences on the kinetics of a small genetic circuit. Finally, we interpret our results in the light of recent in vivo measurements of mean and variability of protein numbers in *E. coli*.

## Methods

We use a stochastic model of gene expression [16] that describes transcription, translation, degradation of mRNA and proteins, and multimerization (binding and unbinding of proteins). The model is implemented using a delayed variant [17] of the Stochastic Simulation Algorithm (SSA) [18], which is similar to the original SSA, but allows arbitrary delays before the release of each of the products of a reaction. A reaction product *X *with a delay *τ *is represented by *X*(*τ*).

### Model of gene expression and RNA and protein degradation

Transcription is modeled by:

(1) $$\begin{array}{cc} S\overset{\infty }{\to }S\left(\tau_{S}\right)+M\left(\tau_{S}\right)\;\; & \tau_{S}\sim Gam\left(\alpha_{M},\;\alpha_{M}k_{M}\right) \end{array}$$

where *S *stands for an available transcription start site (TSS) of a gene and *M *stands for the mRNA coded by that gene. In this reaction, *τ_S _*accounts for the duration of the process of transcription, including the finding of a promoter region by an RNA polymerase, the formation of the closed complex at the transcription start site, the open complex formation, and finally, the promoter escape [19] and elongation. Of these, in general, the most rate-limiting steps are the processes of isomerization and open complex formation [1,2].

To model this multi-step process, we set the reaction rate to infinity, which causes the reaction to occur the moment the reactants become available. Given this, the parameter *τ_S _*fully determines the interval between consecutive productions of transcripts. In our implementation, each time this reaction occurs, a value of *τ_S _*is drawn from a gamma distribution with mean of *k_M_*^-1 ^and coefficient of variation (variance over the mean) of $\alpha_{M}^{-1/2}$. With proper parameter values, the gamma distribution well approximates recent live cell measurements of intervals between productions of consecutive RNAs in *E. coli *[13,20]. We fitted the measurements of time intervals in [20] with the three-exponential model proposed in that work, and with a gamma distribution. The latter results in (*α_M_*, (*α_M _k_M_*)^-1^) of (2.27183, 1070.57) and (2.51171, 565.956) for the low and medium induction levels, respectively. The gamma fits have slightly higher likelihood than the three-exponential ones, so the fit is better.

According to this model, the transcript is released at the same time as the promoter region becomes unoccupied. This approximation assumes that the elongation time is negligible, which relies on observation that the durations of the closed and the open complex formations (in the order of 10^3 ^s) [1,2,13,20] are much longer than elongation (in the order of 10^1 ^or 10^2 ^s) [21,22]. Moreover, in prokaryotes, translation is coupled to transcription [23], and can initiate as soon as the ribosome binding site region (RBS) of the RNA is formed (Shine-Dalgarno sequence) [24]. Consequently, the RNA is available for translation very soon after the RNA polymerase escapes the promoter region.

The RNA, once assembled, is subject to degradation, which we chose to model as a first-order reaction (due to a lack of evidence of degradation mechanisms that depend on, for example, RNA abundance or sequence [25]):

(2) $$M\overset{d_{M}}{\to }\;\emptyset$$

where *d_M _*^-1 ^is the mean mRNA lifetime.

In this model, the degree of noise on the RNA production kinetics can be tuned by varying *α_M_*. Setting *α_M _= *1 yields Poisson distributed mRNA numbers *M *~ Poi(*k_M _d_M_*^-1^), while *α_M _*> 1 and *α_M _*< 1 result in sub- and super-Poissonian distributions of RNA numbers, respectively (both of which have been reported in *E. coli *[6,20]). We note that, for integer values of *α_M_*, the parameter has a physical interpretation: namely, it represents a sequential process with *α_M _*elementary steps, each of duration (*α_M _k_M_*)^-1^, which is in accordance with a sequential process of transcription initiation [1]. However, the best fit is typically obtained for non-integer values of *α_M_*, which do not have a simple physical interpretation. One possible explanation is that the steps have unequal durations or that a step has non-exponential duration (e.g. the open complex formation that involves structural changes of the DNA). Similarly, super-Poissonian RNA dynamics [6] (*α_M _*< 1) require the existence of some additional mechanisms, such as a two-state model of transcription [26].

Translation is modeled by Reaction 3, where *k_P _*is the stochastic rate of translation initiation and *M *is the number of available RNA molecules [27].

(3) $$\emptyset \overset{k_{P}\;M}{\to }P\left(\tau_{P}\right)$$

where *P *is protein and *τ_P _*is the time it takes for the protein to be folded and activated, after translation is complete.

In the simulations, *τ_P _*was set to zero for simplicity, in models of single gene expression, this parameter only shifts the protein numbers in time. If this delay is taken to be a random variable, it also results in increased fluctuations of the protein numbers. For the long-term behavior the time-shift is irrelevant, and the estimations of the contribution to noise are considerable smaller than those from other sources [28]. We tested adding such noise (by setting *τ_P _*to follow a normally distributed delay) and found no qualitative differences in our conclusions.

Finally, the degradation of proteins is modeled via Reaction 4, a first order process [6]. (The rate of protein degradation has been observed to be constant, and identical in different growth conditions [29].)

(4) $$P\overset{d_{P}}{\to }\emptyset$$

where *d_P_*^-1 ^is the mean protein lifetime.

### Modeling the multimerization process

In the case of homomers we consider multiple levels of multimerization (e.g. monomers, dimers, trimers), while for heteromers we only consider second-order multimers, i.e. heterodimers. Note that, in the case of heteromers, the production of each of the two monomers is driven by a different promoter, while in the case of homomers, we assume that there is only one promoter driving the expression.

Heterodimerization and the reverse of this process (which can occur by dissociation or degradation) is modeled by the following reactions:

(5) $$P_{1}+P_{2}\overset{a_{1,2}}{\to }P_{1,2}$$

(6) $$P_{1,2}\overset{u_{1,2}}{\to }P_{1}+P_{2}$$

(7) $$P_{1,2}\overset{d_{P_{1}}}{\to }P_{2}$$

(8) $$P_{1,2}\overset{d_{P_{2}}}{\to }P_{1}$$

where *P*_1 _and *P*_2 _represent the monomers that form the heterodimer *P*_1,2_, when bound to one another. Reactions 5 and 6 model the association and disassociation of monomers, respectively, with *a*_1,2 _being the rate of association and *u*_1,2 _the rate of disassociation. Reactions 7 and 8 model the degradation of monomers *P*_1 _and *P*_2_, respectively, while in the dimeric form. We denote the number of proteins *i *in either monomeric (*P_i_*) or dimeric form (*P_i,j_*) by *X_i _*= *P_i _*+ *P_i,j_*.

The process of production of homomers of order *N *is modeled as follows:

(9) $$\begin{array}{cc} 2\leq n\leq N,\;k\leq n/2:\;\; & P_{i\;\times \;k}+P_{i\;\times \left(n\;-\;k\right)}\overset{a_{i\;\times k,\;i\;\times \;\left(n\;-\;k\right)}}{\underset{}{\to }}P_{i\;\times \;n} \end{array}$$

(10) $$\begin{array}{cc} 2\leq n\leq N,\;k\leq n/2:\;\;\;\;\; & P_{i\;\times \;n}\overset{u_{i\;\times \;k,\;i\;\times \;\left(n\;-\;k\right)}}{\underset{}{\to }}P_{i\;\times \;k}+P_{i\;\times \;\left(n\;-\;k\right)} \end{array}$$

(11) $$\begin{array}{cc} \;2\leq n\;\leq N:\;\; & P_{i\;\times \;n}\overset{n{d_{P}}_{i}}{\underset{}{\to }}P_{i\;\times \;\left(n\;-\;1\right)} \end{array}$$

where *P_i×k _*denotes $\underset{k}{\underset{︸}{P_{i,\cdots \;,i}}}$, the *k*th order homomer of proteins *P_i_*. Reactions 9 represent the association of an order-*k *homomer and an order-(*n - k*) homomer to form an order-*n *homomer, while Reactions 10 represent the reverse process. Reactions 11 represents the degradation of any of the *n *proteins that are part of an order-*n *homomer, resulting in an order-(*n - *1) homomer. The rates *a_i×k,i×_*_(_*_n-k_*_)_and *u_i×k,i__×_*_(_*_n-k_*_)_, are the association and disassociation rates for the combinations of different order homomers, and $d_{P_{i}}$ is the protein degradation rate. We define $X_{i}≐\sum_{k=1}^{N}kP_{i\times k}$, as the total number of proteins in the system, regardless of their form. This definition is analogous to that of *X*_1 _and *X*_2 _in the heterodimer model.

### Toggle switch

We model a genetic toggle switch [30], which consists of two genes, expressing proteins *P*_1 _and *P*_2_, respectively. The protein expressed by the first gene inhibits the expression of the second gene, whose protein product in turn inhibits the expression of the first gene. Interactions between repressor proteins and promoters are implemented by assigning the rate *k_M _*in Reaction 1 to be a function of the number of repressor molecules present in the system, as follows:

(12) $$k_{M_{j}}={\left(1+{K_{i,j}}^{-1}P_{i\times n}\right)}^{-1}k_{{M_{j}}^{\prime }}$$

where *P_i×n _*is the order-*n *multimer of gene *i*, *K_i,j _*is the disassociation constant for the multimer binding to the promoter of gene *j*, and ${k_{M_{i}}}^{\prime }$ and $k_{M_{j}}$ are the maximal and effective transcription rates of the *j*th gene, respectively. (Here (*i*, *j*) = (1, 2) or (*i*, *j*) = (2, 1).)

## Results

All models and simulations were performed using the simulator SGNS2 [31]. The following description of parameter selection applies to all simulations, unless otherwise mentioned. The protein degradation rate *d_P _*is set to unity. This reduces the dimension of the parameter space: rate constants and time delays are expressed in units of protein lifetime. The parameters *d_M_*, *k_M_*, and *k_P _*are varied logarithmically within the range [10^−1^, 10^1^], *α_M _*is varied in the set {1, 2, 3, 5, 10}. Each of the parameters is varied independently. Variation in the parameter values within these ranges leads to significant variation in protein abundance (e.g. a range of 10^6 ^in the mean protein level).

To quantify changes in mean and noise levels when comparing models, we define "gain" as the ratio of the value of the tested model to that of the null model. Gains above unity imply that the tested model exhibits values larger than the null model, while gains less than unity imply the opposite.

For simplicity, the multimerization association rates *a_i×n,i_*_×(_*_n-k_*_)_ are assumed to be infinite, while the disassociation rates *u_i×n,i×_*_(_*_n-k_*_)_ are set to zero, this does not affect our results qualitatively, and facilitates comparison between models. This issue is further discussed in the results section. Finally, we run each simulation for 10^5 ^time units so that the system spends most of the time near equilibrium. We sample the state of the system (all molecules numbers) with intervals of one time unit.

Note that we include the transient in the samples as we sample from time zero. This is due to the fact that the system does not reach an equilibrium in a finite time interval. From observations of the time series we found that, for a duration of 10^5 ^time units, the systems is, for more than 99*.*9% of time, close to equilibrium. That is, given 10^3 ^simulations, if one extracts samples of multimer numbers from this region, one cannot distinguish them, in a statistical sense, from the samples of the distribution of multimer numbers at the last time moment.

### Homodimers

We compared the mean levels of a monomeric protein (*X*_1_) and of a homogeneous dimer (*P*_1,1_). The two models are taken to be identical except for the dimerization. Since the expression rates are identical, the mean level of the dimer must be less than or equal to half the mean level of monomer. We consider two cases for the dimer model: one in which only the dimer is functional, and one in which both the monomer and dimer are functional. In the latter case, we asses the joint dynamics of both the monomeric (*P*_1_) and dimeric (*P*_1,1_) forms. In this case, the amount of functional proteins is given by $Y_{1}≐P_{1}+P_{1,1}$.

**Figure 1:**
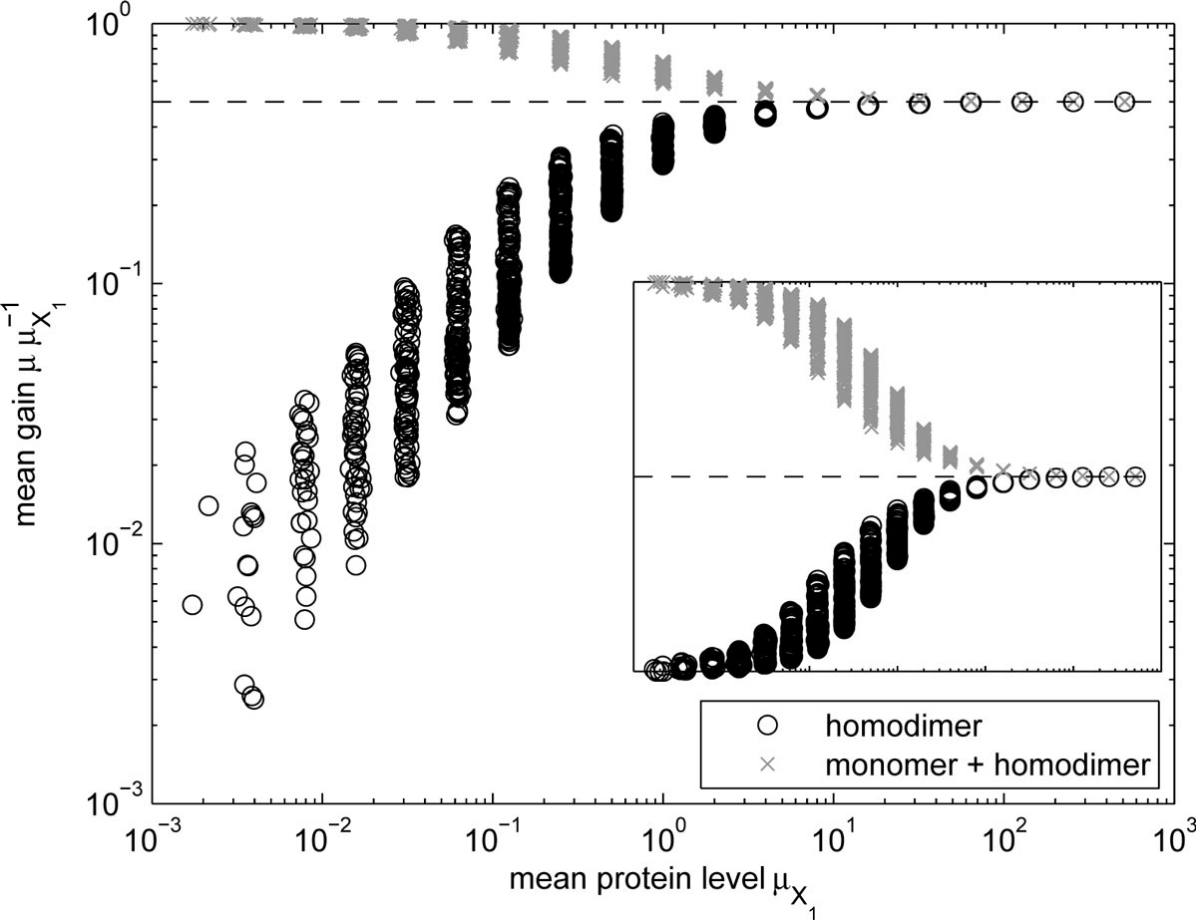
**Change in mean levels due to homodimerization**Relative mean levels of homodimers *P*_1,1 _and the total number of molecules $Y_{1}≐P_{1}+P_{1,1}$ as a function of the mean monomer level $\mu_{X_{1}}$. The dashed line indicates a gain of one half. The inset shows linear gain.

We simulated the models with several parameters values of *d_M_*, *α_M_*, *k_M_*, and *k_P _*as described above. Taking *μ *as the mean level of the molecules of interest and $\mu_{X_{1}}$as the mean level in the monomeric model, the ratio $\mu \mu_{X_{1}}^{-1}$ is plotted as a function of $\mu_{X_{1}}$in Figure 1. (The mean $\mu_{X_{1}}$is determined by *d_M_*, *k_M_*, and *k_P_*).

From Figure 1, we observe that for high values of $\mu_{X_{1}}$, the mean level of the homodimers *P*_1,1_, is half that of *X*_1_, while for low values of $\mu_{X_{1}}$ it approaches zero, because it is more probable that there is a single protein in the system, precluding the formation of a dimer. The total number of monomers and dimers (*Y*_1_) varies in an inverse fashion to that of dimers alone, since *Y*_1 _= *X*_1 _*- P*_1,1 _(cf. inset in Figure 1).

The points in Figure 1, while each being resultant from a unique set of parameter values, are grouped into bands. This is due to the fact that various combinations of parameter values result in identical mean levels but differing noise levels. The changes due to varying individual parameter values can be explained as follows. The expected mean protein level is determined by *k_M _d_M_*^−1 ^*k_P _d_P_*^−1^, while the noise is increased with the inverse of the mean and the inverse of *α_M_*, in an intricate manner (see [32] for an approximation). It follows that increasing (decreasing) *k_M _*or *k_P _*or decreasing (increasing) *d_P _*will result in an increase (decrease) in the protein mean level (x-axis) and a consequent increase (decrease) in the mean gain (y-axis), and increasing (decreasing) *α_M _*will have no effect on the protein mean (x-axis) and will decrease (increase) the mean gain (y-axis).

**Figure 2:**
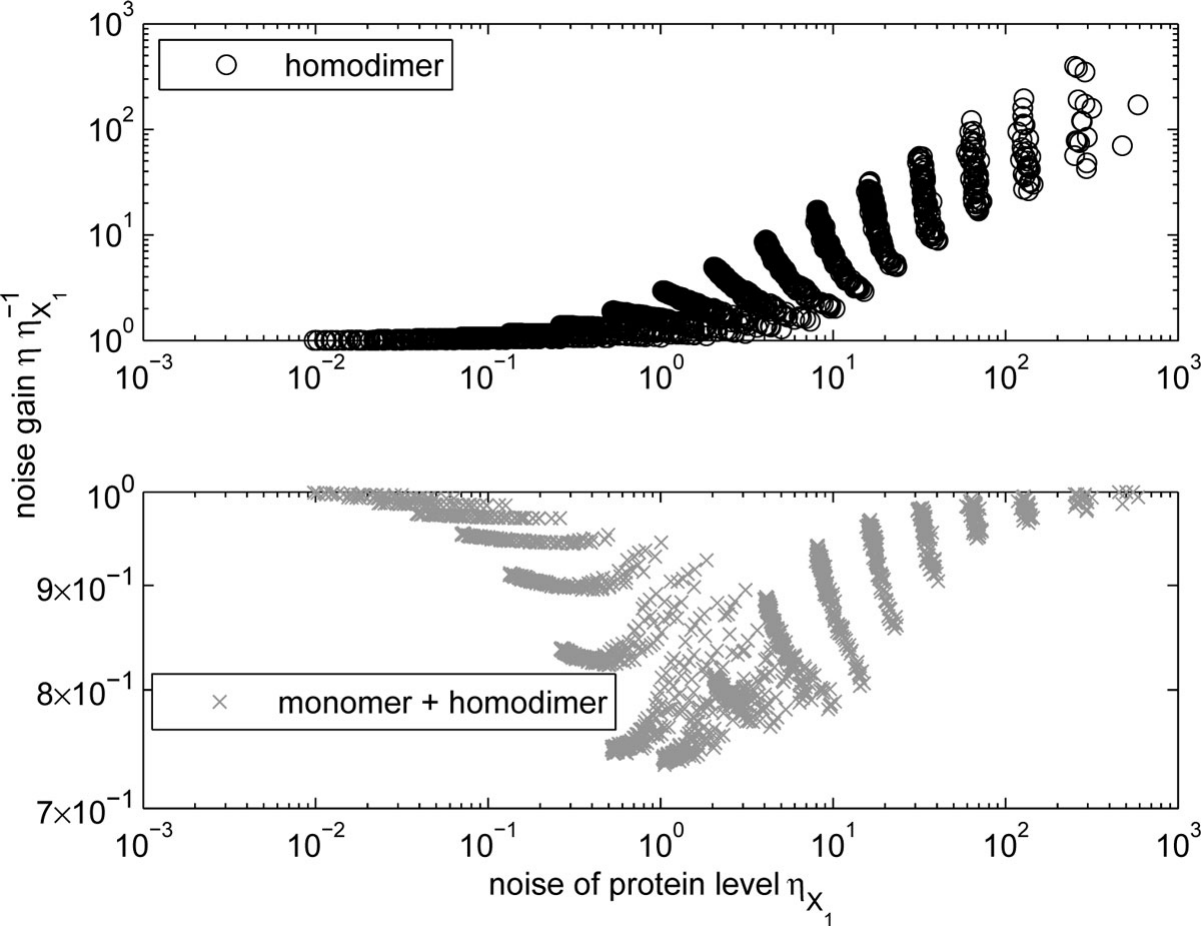
**Change in noise levels due to homodimerization**. Relative noise levels of homodimers *P*_1,1 _(upper panel) and the total number of molecules $Y_{1}≐P_{1}+P_{1,1}$ (lower panel) as a function of the noise level of monomers $\eta_{X_{1}}$.

Next, using the same models, we compared the noise levels, quantified by the square of the coefficient of variation, denoted by *η*. The results are shown in Figure 2. When comparing with Figure 1, it is important to note that, in general, models with low and high noise levels correspond to the models with high and low mean levels, respectively. This relationship holds for low mean levels, for which the low-copy number noise dominates, whereas for high mean levels other parameters dominate the noise. The points corresponding to simulations with identical mean levels of *X*_1 _are again contiguous, but they do not form vertical lines.

Given the properties of the model, the noise in the homodimer numbers (*P*_1,1_) is always greater than that of the non-dimerizing proteins (*X*_1_). For parametrizations which yield a dimer level equal half of the number of protein units in the system (i.e. right hand side of Figure 1) the noise gain is equal to unity, implying no increase in noise due to the dimerization process. However, further decreases in the mean levels lead to significant increase in the noise (gains of the order of 10^2^, as seen in the upper panel of Figure 2).

The lower panel of Figure 2 indicates that the noise in the total number of molecules (*Y*_1_) is always smaller than that of the monomers. In the two extremes, the total number consists entirely of monomeric or dimeric forms of the protein, so the noise level of the functional proteins must match that of a single form. However, when the numbers of the monomeric and dimeric form are balanced, the noise level of the functional molecules (*Y*_1_) is slightly suppressed by the dimerization when compared to *X*_1_.

It is possible to see that the choice of multimerization association rates *a_i×n,i×_*_(_*_n−k_*_)_and disassociation rates *u_i×n,i×_*_(_*_n−k_*_)_ does not affect the above results qualitatively. Any other settings will inevitably lower the number of dimers. Thus, the conclusion that dimer numbers must be lower than half the number of monomers holds. Also, the number of monomers and dimers (*Y*_1_) will increase, since they will still follow the relationship *Y*_1 _= *X*_1 _*− P*_1,1_. Additionally, the noise in dimer numbers will increase due to the low-copy number effect, and consequently, the conclusion that the noise must be above unity holds. Finally, the noise in the numbers of monomers and dimers will become more similar to that of the monomers alone (resulting in a noise gain closer to unity).

### Heterodimers

Next, we consider a scenario in which a dimer is formed by the protein products *P*_1 _and *P*_2 _of two distinct genes. For simplicity, the kinetics of protein production are assumed to be identical for *P*_1 _and *P*_2_. We compared the behaviour of this heterodimer with a corresponding homodimer model. (Alternatively, one could consider a model in which a single promoter controls the expression of *P*_1 _and *P*_2_. We opted not to investigate this case, because the kinetics would lie somewhere between the homodimer case and the heterodimer case described above). For the purposes of comparison, the mRNA production rate *k_M _*is doubled in the homodimer, to compensate for the existence of two genes (each expressing at rate of *k_M_*) producing the components of the heterodimer.

**Figure 3:**
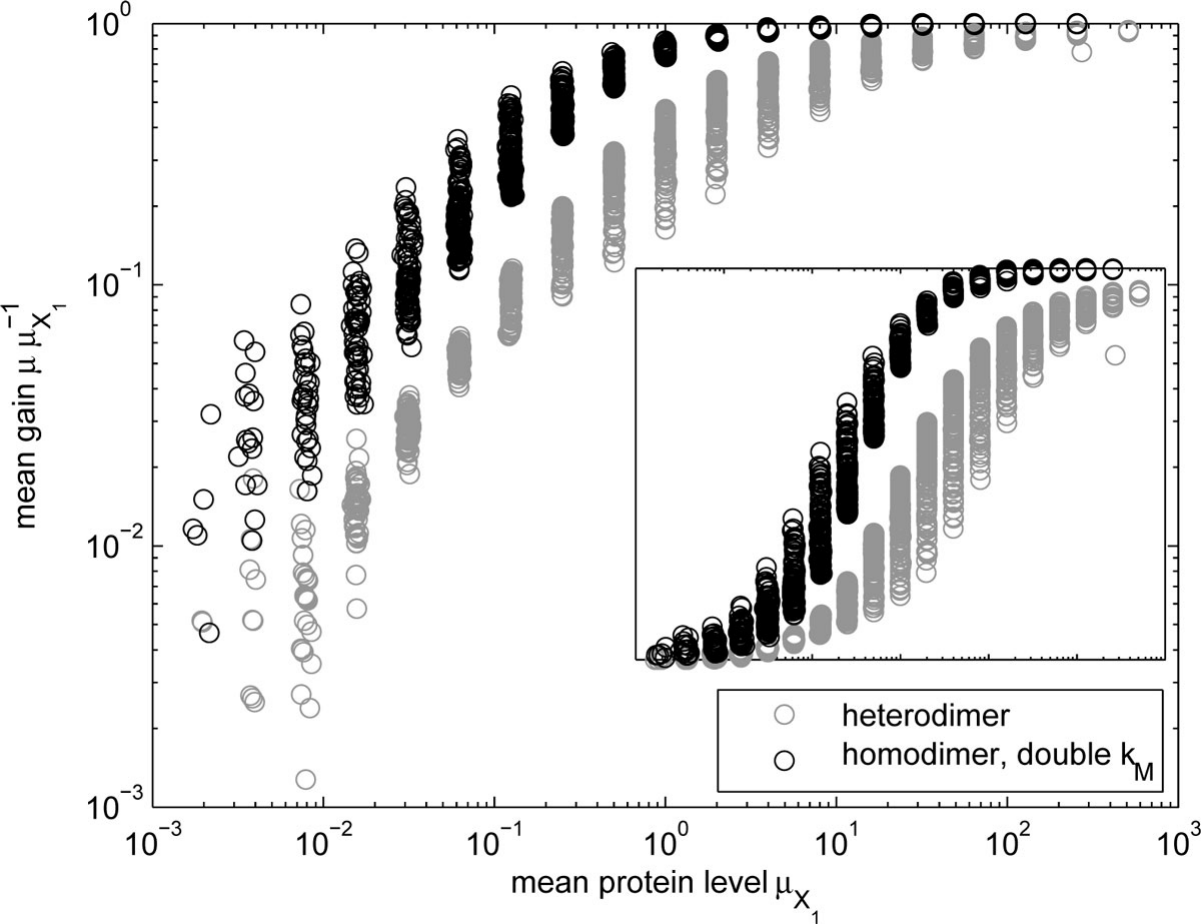
**Change in mean levels due to heterodimerization**. Relative mean levels of heterodimers *P*_1,2 _and homodimers *P*_1,1 _(with double *k_M _*to compensate for the reduction in the mean level) as a function of the mean monomer level $\mu_{X_{1}}$. The inset shows linear gain.

The ratio of the mean levels of the heterodimer and homodimer is plotted in Figure 3 as a function of the mean level of one of the proteins (*X*_1_, or equivalently *X*_2_). As in the homodimer case, when the mean levels is high, nearly all proteins are present in dimeric form, and so both models have the same mean abundance of functional protein, whereas for low means, there is a population of unpaired proteins which results in a reduction of the mean level of the dimer when compared to the non-dimerizing gene. Moreover, the heterodimer case exhibits greater reductions in the mean than the homodimer case, since to form a dimer, the "missing" protein has to be of a certain type.

**Figure 4:**
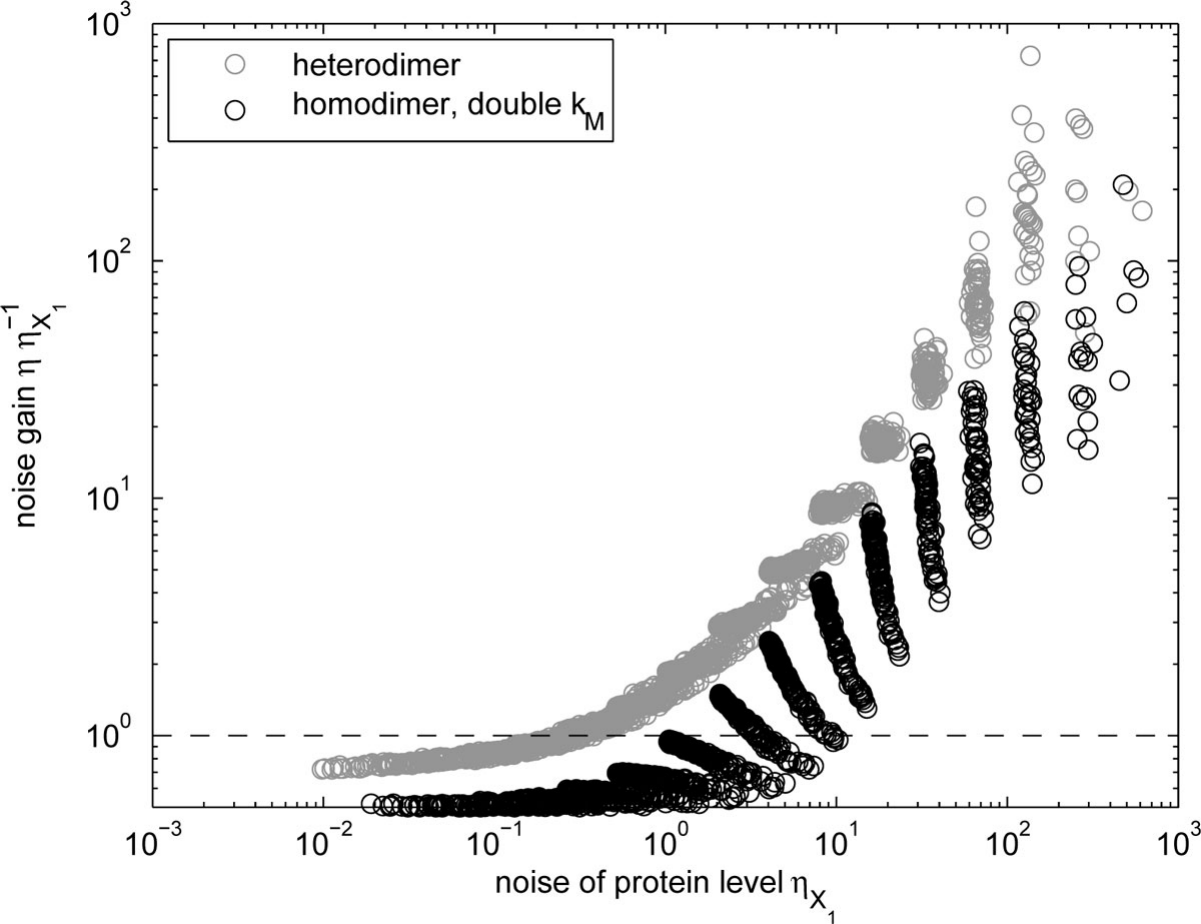
**Change in noise levels due to heterodimerization**. Relative noise levels of heterodimers *P*_1,2 _and homodimers *P*_1,1 _(with double *k_M _*to compensate for the reduction in the mean level) as a function of the noise level of monomers *X*_1_. The dashed line indicates a gain of unity.

We also studied the ratio of the noise levels of the above models, as presented in Figure 4. The noise levels exhibit a behavior similar to the homodimer case presented previously (cf. Figure 2), but since in the present case the mean level is not halved (due to the increased transcription rate *k_M_*) the noise gain can be decreased below unity. Specifically, for high mean levels in the homodimer, the noise is suppressed to one half, essentially due to the doubled transcription rate, in this case the dimerization process does not introduce much noise (noise gain equals unity, Figure 2). On the other hand, for low mean levels, the results follow those presented earlier. That is, the greater decrease of mean numbers results in higher gain in noise levels. Moreover, we find that the noise suppression ability of the heterodimeric form is less than that of the homodimer, due to the weaker temporal correlation between the numbers of the two distinct dimer-forming proteins.

### Higher-order multimers

Finally, we studied if and how the results generalize for higher-order multimers. Since the effects were more prominent in homodimers, we studied only multimers of homogeneous proteins of increasing order. We present results for homomers of orders *N *∈ {2, 3, 4, 5} (dimer, trimer, tetramer, and pentamer, respectively). We also tested for decamers (*N *= 10) (data not shown) as an extreme case, and found the qualitative results to agree with those presented here.

Analogous to the homodimeric case (Figure 1), the mean levels of order-*N *homomers are subject to gains of at most *N*^−1^. In addition, as *N *increases, there is an ever increasing probability of lacking the necessary components to form the multimers, so the values of gain are generally lower than *N*^−1^. We note that even models with mean levels of proteins in the order 10^3 ^are subject to significant losses in the multimerization procedure as the order is increased (e.g. *N *= 5). Likewise, the noise gain follows the trend shown earlier in Figure 2, with higher-order homomers being more noisy.

**Figure 5:**
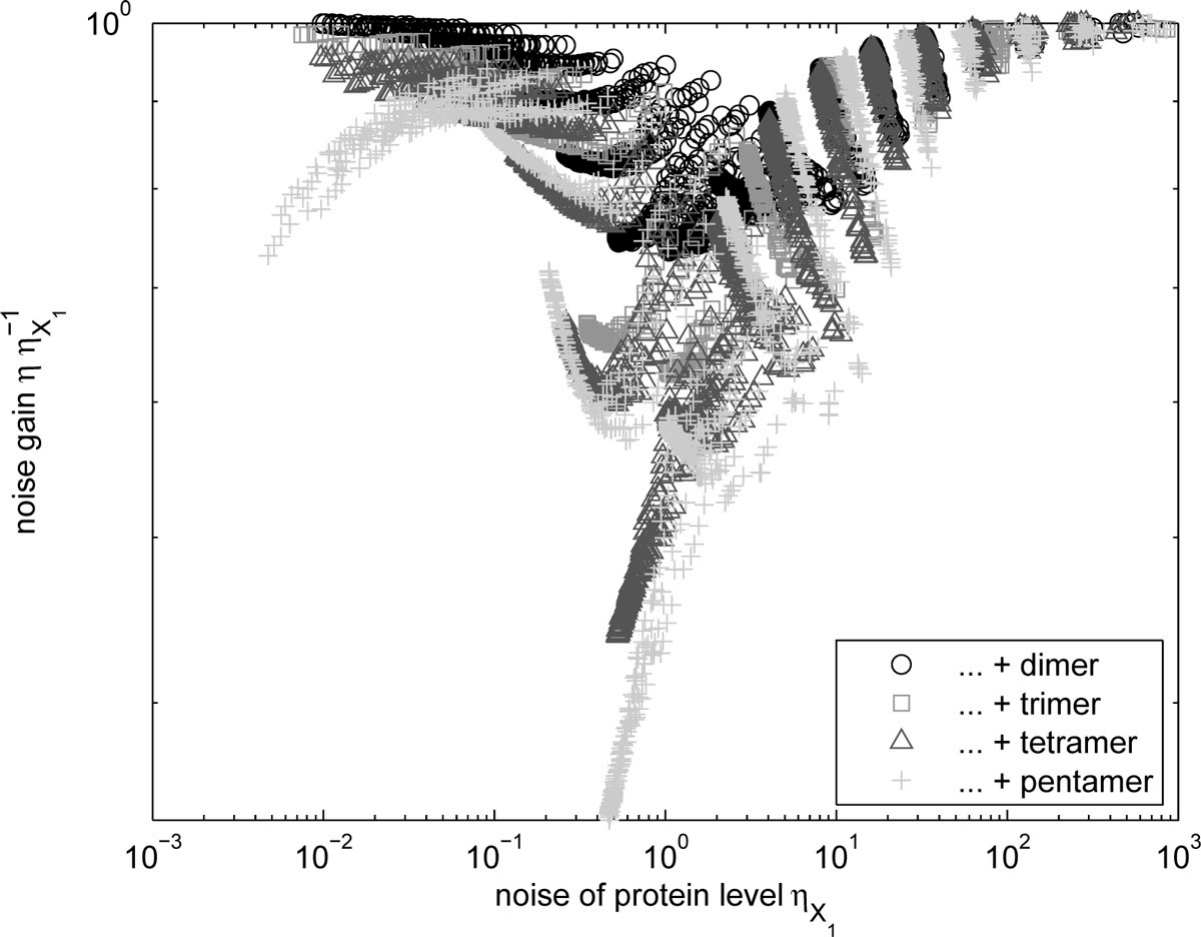
**Change in noise levels of total number of molecules due to higher-order multimerization**. Relative noise levels of total number of molecules $Y_{1}≐P_{1}+\cdots +P_{1\times N}$ with multimerization of different orders as a function of the noise level of monomers *X*_1_.

In Figure 5, we show the noise in homomerization in the case where all protein forms (*P*_1_*, ⋯, P*_1×*N*_) are functional. Here, the results agree with the dimer case (see the lower panel of Figure 2). Higher-order multimerization can exhibit greater noise suppression capabilities, but only for a more limited range of parameter values that lead to properly balanced numbers of the multimers in the various forms.

**Figure 6:**
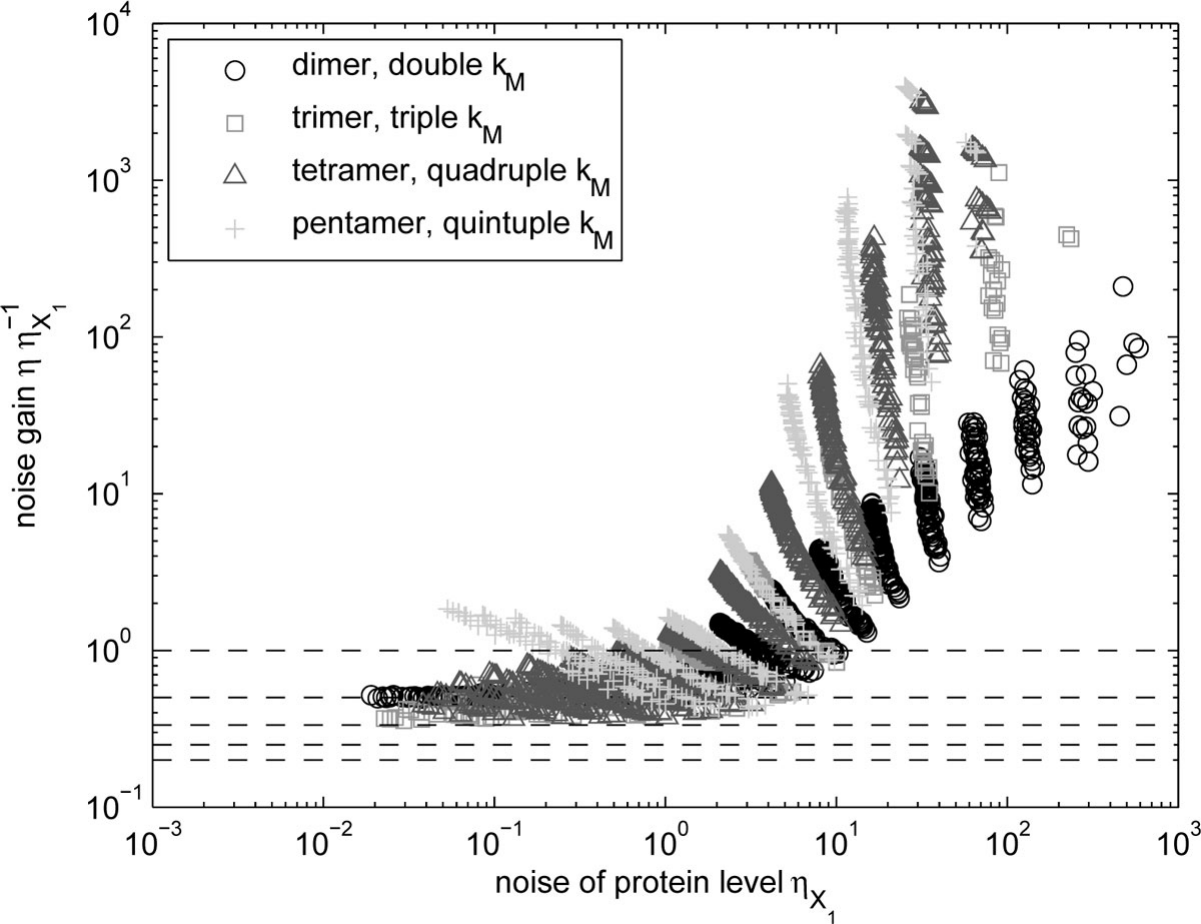
**Change in noise levels of homomers due to higher-order multimerization**. Relative noise levels of homomers (with adjusted *k_M _*to compensate for the reduction in the mean level) with multimerization of different orders as a function of the noise level of monomers $\eta_{X_{1}}$. The dashed lines indicate gains of unity, one half, one third, one quarter, and one fifth.

We also compared noise levels of strictly monomeric proteins to those of multimers. For this comparison, the transcription rate of the proteins composing the multimers are chosen so that the mean numbers of the multimer form are similar to those of the strict monomer. The results (Figure 6) are similar to the homodimer case (Figure 4). Potentially, this scheme allows the noise level to be suppressed to *N*^−1^th of the original value, but this is only achievable for highly expressed genes. In general, higher-order multimerization can only lead to noise suppression within a limited range of parameter values. More specifically, in the case of high order multimers, the fluctuations in protein numbers alone determines if the noise in multimer numbers is amplified or suppressed.

### Temporal regulation of the number of multimers

In organisms such as bacteria, regulation of gene expression is performed mostly at the stage of transcription initiation, at the promoter region. Consequently, temporal variability in monomer levels is strongly controlled by factors regulating transcription initiation. However, the production of multimeric proteins involves an additional stochastic process - multimer formation itself. As a result, one expects that a mechanism operating at the stage of transcription initiation may exhibit reduced control over the temporal numbers of multimer, as compared with proteins that function as monomers. This may pose limits on the selection of higher-order multimers.

We studied how the process of multimerization affects the ability to regulate multimer numbers via the regulation of the kinetics of production of the monomers alone. We hypothesize that the optimal design would have the multimer numbers following the monomer numbers as closely as possible. That is, the cross-correlation between the numbers of monomers and multimers should be unity at zero-lag, the lag referring to the time-shift in the series of the two numbers for which the correlation is evaluated. This cross-correlation should also decay as quickly as possible with lag, because otherwise the correlation with past events would make it difficult for the system to respond to current changes.

We found that, in general, the cross-correlation functions estimated from our simulations exhibited maximal correlation at zero-lag. We thus use the cross-correlation at zero-lag to quantify the loss in control due to the multimerization process. To study the decay of the cross-correlation in each model, we estimate the point in lag where the cross-correlation attains a value that is half of the maximum, denoted by half-life of the protein-homomer cross-correlation. We note that, for an exponential decay of correlation, this half-life would equal ln 2 times the mean response time. However, since the decays measured are not purely exponential, but rather combinations of several exponentially decaying terms, the half-life only reflects the response times in a qualitative sense.

**Figure 7:**
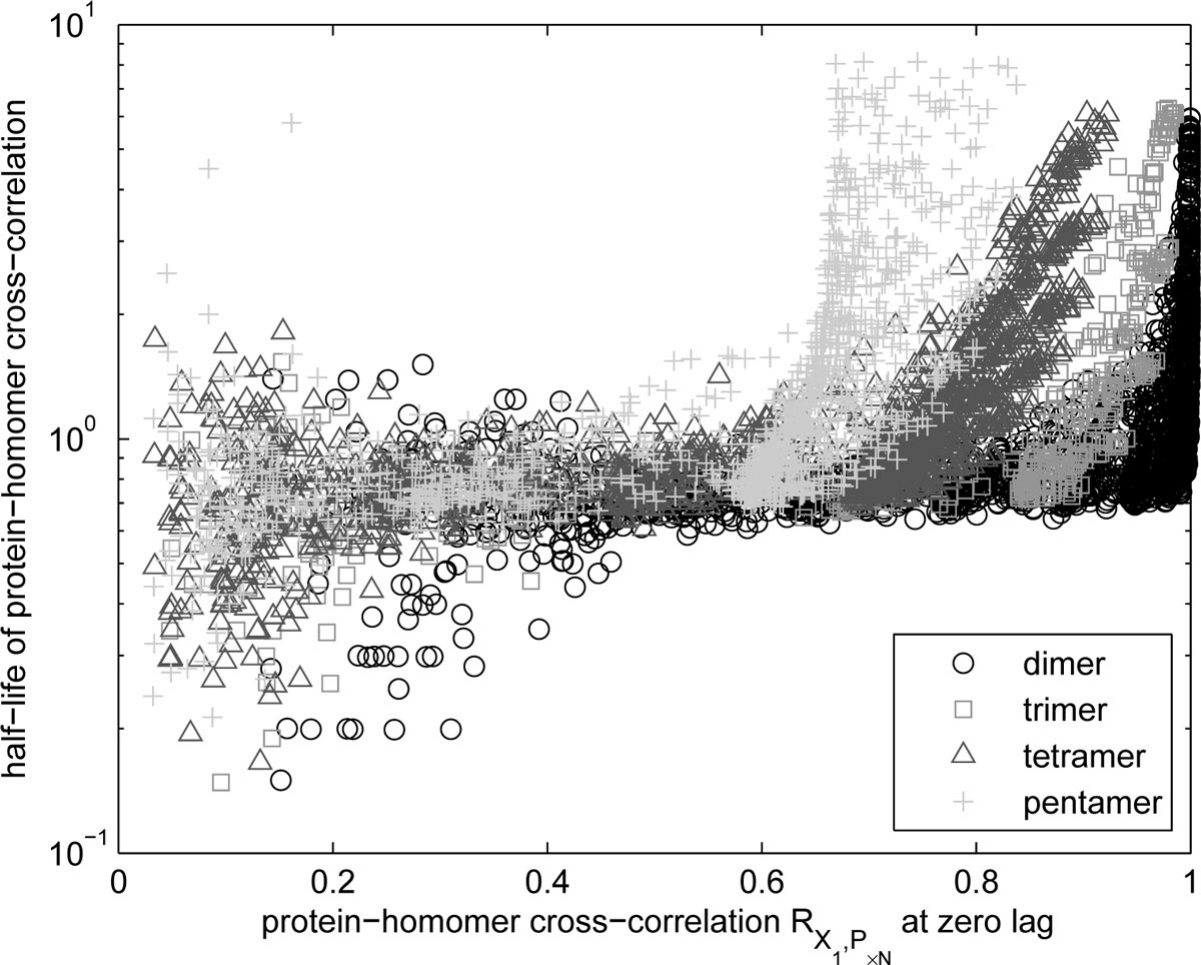
**Cross-correlation between homomers and monomers**. Half-life (in units of protein lifetime) of cross-correlation between homomer and monomer levels as a function of the zero-lag (maximum) cross-correlation between the two.

To assess these quantities, we sampled the state of the models with intervals of 1*/*10 of one time unit, and ran the simulations to obtain 10^5 ^samples. For each multimer order, we compared the half-life of the protein-homomer cross-correlation with the cross-correlation at zero-lag (Figure 7). The results indicate that for higher orders of multimerization, there is a loss in correlation in the homomers, when the value of the correlation was high. The results indicate that as the order of multimerization increases, the correlation at zero lag of the homomers decreases. This is only significant if these homomers had high cross-correlation to begin with. Moreover, in general, high correlations imply higher half-lives regardless of the order of the multimer, which indicates that the multimers cannot exhibit high control and fast regulation at the same time. Also generally, for multimers with a specific value of correlation at zero lag, lower order multimers will have shorter response times.

### Genome wide assessment of cell-to-cell variability and degree of multimerization in Escherichia coli

In [6], genome-wide data was collected on the mean and standard deviation of protein copy numbers in populations of *E. coli *under optimal growth conditions, for large sets of both essential and non-essential genes. (Essentiality of a gene is defined according to the following criteria (http://www.shigen.nig.ac.jp/ecoli/pec/index.jsp): in general, genes for which lethal mutants have been isolated are classified as essential.) From the EcoCyc database (http://www.ecocyc.org/) for the strain *E. coli *K-12 MG1655, we assessed which of these proteins form multimers and, if so, how many subunits of each gene is involved in the multimer.

Table 1 presents the fraction of proteins that form each of the various orders of multimers, for both essential and non-essential genes. Also, for each order we computed the median (med *μ*) of the mean protein numbers and the median of the squared coefficient of variation (med *η*) of protein numbers in individual cells. Note that the mean and noise levels are extracted from observation of individuals proteins alone, rather than proteins in multimeric form.

**Table 1:** Mean and noise in bacterial genes as a function of multimerization noise.

		number of homogeneous subunits									
		
		1	2	3	4	5	6	7	8	9	10
essential	% genes	54.55	29.75	4.13	4.96	0.00	4.96	0.83	0.00	0.00	0.83
	
	med *μ*	33.82	58.21	36.76	72.66	n/a	110.05	2150.20	n/a	n/a	17.07
	
	med *η*	0.15	0.14	0.19	0.14	n/a	0.15	0.13	n/a	n/a	0.80

non-essential	% genes	72.80	18.06	1.23	4.57	0.22	1.78	0.00	0.00	0.00	1.00
	
	med *μ*	8.84	16.12	79.14	27.72	74.27	54.54	n/a	n/a	n/a	8.88
	
	med *η*	0.26	0.21	0.16	0.20	1.16	0.19	n/a	n/a	n/a	0.23

Fraction of genes, their mean expression level *μ*, and noise level *η *for essential and non-essential genes, which are known to form different orders of multimers for function.

In general, essential genes exhibit higher mean levels than non-essential ones. Also, their noise levels appear to be somewhat constant [6]. Further, proteins from essential genes appear to form higher-order functional units, and the mean levels of proteins forming high-order multimers are much higher. Non-essential genes also exhibit higher mean levels of protein numbers when forming high-order multimers.

In [6], it is also suggested that the protein numbers of essential genes lie on a noise floor. This floor was hypothesized to originate from fluctuations in cellular components (e.g. metabolites, polymerases, ribosomes) [6]. Our results above suggest that multimerization should offer a means to reduce the copy number noise level of proteins in the functional form below this noise floor. If so, one would expect the protein products of essential genes to have a greater tendency for multimerization than non-essential ones, since they already lie on the noise floor in the monomeric form while the latter should be able to select for reduced noise by other means, such as tuning the noise in the process of RNA production. The data in the EcoCyc database agrees with this prediction. Further, for this strategy of noise reduction in essential genes to be successful, one would expect to observe also much higher mean protein numbers in the case of highly multimerizing genes. This is also confirmed by the data in Table 1.

### Toggle switch

Finally, we tested if multimerization can affect the stochastic behavior of genetic circuits. To this end, we simulated models of genetic toggle switches [30], using homomers of different order (*N *∈ {1, 2, 3}) as regulatory molecules. We then measured the mean switching times of each model, that is, the average time the switch spends on one of the two states (either *P*_1*×N *_*< P*_2*×N *_or *P*_1*×N *_*> P*_2*×N*_). To account for the fact that the mean switching time is sensitive to the mean protein levels, the dimer and the trimer were simulated with double and triple *k_M_*, respectively (to provide similar mean level of the regulatory molecules in the different models).

**Figure 8:**
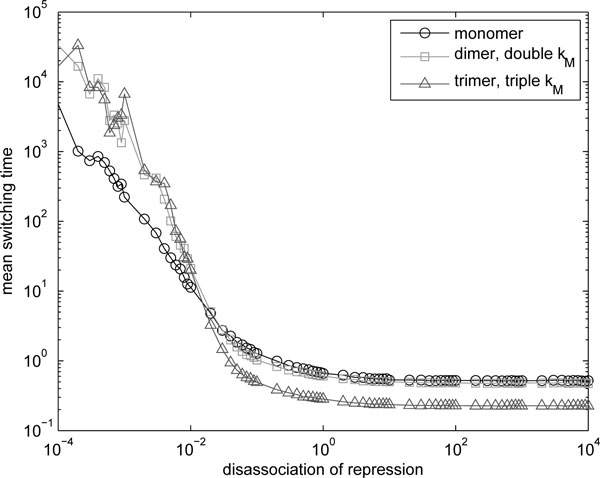
**Change in mean switching time of toggle switch due to multimerization**. Mean switching time of a toggle switch as a function of the inverse repression strength *C*, where the genetic interactions are implement with different orders of homomers, as a function of disassociation strength of repression.

The parameters used in the models were: RNA degradation rate *d_M _*= 6 *d_P_*, expected transcript number *k_M _N*^−1^*d_M_*^−1 ^= 5, transcription kinetics shape *α_M _*= 1, expected number of protein per RNA *k_P _d_P_*^−1 ^= 5, and disassociation of repression *K *= *C k_M _N*^−1 ^*d_M _^−1 ^k_P _d_P_*^−1^, where *C *was varied in the range [10^−4^, 10^4^] with approximately logarithmic spacing (i.e. {*a *10*^b^| a *∈ {1, 2, 3*, ⋯ *, 9}*, b *∈ {*−*4*, −*3*, −*2*, ⋯ *, 4}}). The gene expression parameters are in agreement with live cell measurements in *E. coli *[6]. The switch's state was sampled with intervals of 1*/*30 time units, the simulations provided 10^6 ^samples. The mean switching time as a function of the inverse of the repression strength *C *is shown in Figure 8.

In Figure 8 it is visible that the mean switching time is different for different orders of homomerization. In the region where the repression is strong, the multimerization results in increased switching times. On the other hand, for low repression strength, the mean switching time is decreased for the homomers. In general, higher-order multimerization appears to offer a wider range of mean switching times for the toggle switch. These differences in the kinetics of the models are due to the differences in noise levels of the functional multimers of different orders, confirming thus that the order of multimerization has a tangible effect on the kinetics of genetic circuits.

## Conclusion

We studied how the order and nature of the multimerization of a protein affects the temporal variability in copy number. We found that multimerization increases noise, in that it necessarily reduces the numbers of functional protein complexes. However, if both monomers and dimers (or higher-order multimers) are functional, the dimerization process suppresses noise in the numbers of functional complexes, for a range of parameter values for which dimers and monomers are present in similar amounts. Alternatively, if the introduction of a multimerization process is combined with an increase of transcription rates to compensate for the decrease in number of functional complexes, then multimerization can also lead to a reduction of noise levels on the numbers of these functional complexes. The same holds true for heterodimers, but the noise suppression is less significant because the production of the subunits is less coordinated.

In addition, multimerization reduces the degree of control exerted by gene regulatory mechanisms on the copy number of functional complexes. Compensatory increases in this control, which are constrained by the noise introduced by the multimerization process, will necessarily lead to an increase on the mean response time of the gene.

Finally, the stochastic effects of multimerization were found to propagate to the level of genetic circuits, further supporting the notion that this process is likely under selection pressure for reasons other than functionality of proteins: namely, for their effects on the dynamics of protein numbers and on the dynamics of genetic circuits. This selective pressure may be confirmed by future studies, but the observation that essential genes (whose numbers of the proteins in monomeric form alone lie on the noise floor) are more likely to multimerize than non-essential ones, is already tentative evidence for the existence of this pressure.

## Competing interests

The authors declare that they have no competing interests.

## Authors' contributions

ASR, BI, and AH conceived the study. ASR supervised the interpretation of data. AH and HT performed the modeling and analysis. All authors performed research. AH and ASR drafted the manuscript. The funders had no role in study design, data collection and analysis, decision to publish, or preparation of the manuscript.

## Declarations

The publication costs for this article were funded by the Finnish Funding Agency for Technology and Innovation (grant no. 40226/12).

This article has been published as part of BMC Systems Biology Volume 7 Supplement 1, 2013: Selected articles from the 10th International Workshop on Computational Systems Biology (WCSB) 2013: Systems Biology. The full contents of the supplement are available online at http://www.biomedcentral.com/bmcsystbiol/supplements/7/S1.
